# The Mediterranean Sea Regime Shift at the End of the 1980s, and Intriguing Parallelisms with Other European Basins

**DOI:** 10.1371/journal.pone.0010633

**Published:** 2010-05-19

**Authors:** Alessandra Conversi, Serena Fonda Umani, Tiziana Peluso, Juan Carlos Molinero, Alberto Santojanni, Martin Edwards

**Affiliations:** 1 Marine Sciences Institute (ISMAR), Italian National Research Council (CNR), La Spezia, Italy; 2 Marine Institute, University of Plymouth, Plymouth, United Kingdom; 3 Department of Life Sciences, University of Trieste, Trieste, Italy; 4 Leibniz Institute of Marine Sciences, Kiel, Germany; 5 Marine Sciences Institute (ISMAR), Italian National Research Council (CNR), Ancona, Italy; 6 Sir Alister Hardy Foundation for Ocean Science (SAHFOS), Plymouth, United Kingdom; University of Hull, United Kingdom

## Abstract

**Background:**

Regime shifts are abrupt changes encompassing a multitude of physical properties and ecosystem variables, which lead to new regime conditions. Recent investigations focus on the changes in ecosystem diversity and functioning associated to such shifts. Of particular interest, because of the implication on climate drivers, are shifts that occur synchronously in separated basins.

**Principal Findings:**

In this work we analyze and review long-term records of Mediterranean ecological and hydro-climate variables and find that all point to a synchronous change in the late 1980s. A quantitative synthesis of the literature (including observed oceanic data, models and satellite analyses) shows that these years mark a major change in Mediterranean hydrographic properties, surface circulation, and deep water convection (the Eastern Mediterranean Transient). We provide novel analyses that link local, regional and basin scale hydrological properties with two major indicators of large scale climate, the North Atlantic Oscillation index and the Northern Hemisphere Temperature index, suggesting that the Mediterranean shift is part of a large scale change in the Northern Hemisphere. We provide a simplified scheme of the different effects of climate vs. temperature on pelagic ecosystems.

**Conclusions:**

Our results show that the Mediterranean Sea underwent a major change at the end of the 1980s that encompassed atmospheric, hydrological, and ecological systems, for which it can be considered a regime shift. We further provide evidence that the local hydrography is linked to the larger scale, northern hemisphere climate. These results suggest that the shifts that affected the North, Baltic, Black and Mediterranean (this work) Seas at the end of the 1980s, that have been so far only partly associated, are likely linked as part a northern hemisphere change. These findings bear wide implications for the development of climate change scenarios, as synchronous shifts may provide the key for distinguishing local (i.e., basin) anthropogenic drivers, such as eutrophication or fishing, from larger scale (hemispheric) climate drivers.

## Introduction

Climate variability occurs at a variety of scales, from the rather well-known annual, to the decadal, centennial, and larger millennial (paleoclimatic) scales. The relevance of the decadal scale for marine ecological changes has become evident in the last 30 years, when multi decadal time series of *in situ* collections provided records long enough for patterns to be statistically discernible. In particular, it is at the decadal scale that the link between climate and marine biota variations has been found for a range of marine ecosystems, including different oceanic regions, starting with the initial findings in the 1980s of synchronous changes across trophic levels [1], [2], [3], [4], and later with the findings of covariations between indices of large scale climatic variability and biota in the 1990s and the 2000s [5], [6], [7], [8], .

Abrupt shifts involving both the physical and the ecological systems, called regime shifts [11] have been the focus of recent attention. They hold particular relevance in the marine realm, because they encompass a multitude of physical properties and ecosystem variables, and can involve all trophic levels of marine food webs and the associated biogeochemical cycles. The term “regime shift” in general is used to indicate an extensive and relatively abrupt change, happening within a few years, between contrasting persistent states of a system [12], [13], [14], [15], [11]. The new regime conditions may last a decade or more [16], and, in some cases, there may be no return [17].

In this work we present evidence that an abrupt shift, which encompassed marine circulation, hydrological and biological properties, occurred at the end of the 1980s in the Mediterranean Sea. We also provide novel analyses that suggest that it can be linked to the larger scale, northern hemisphere climate.

This work is divided in four sections as follows: The documentation of long term changes in the trophic web in the Adriatic Sea (eastern Mediterranean) shows a shift at the end of the 1980s (*section a*). Subsequently, we discuss the changes in the north western Mediterranean where an abrupt increase in zooplankton abundance during the same period is seen (*section b*). A review of the Mediterranean Sea physical oceanography literature identifies a basin-wide change around 1987, in both surface circulation and deep water convection, although the two have not been associated so far (*section c*). The merging of the above findings leads us to identify a regime shift in the Mediterranean Sea at the end of the 1980s. We hence provide new analyses of northern hemisphere climate indices and of Mediterranean hydrological properties at different scales, from basin to local, that allow identifying a common period change in the late 1980s (*section d*). Owing to the synchronicity in time with regime shifts reported by several authors in the North, Baltic, and Black Seas, we hypothesize that the Mediterranean Sea regime shift is associated to a larger, northern hemispheric climatic pattern. We then discuss the mechanisms of transmission from large (hemispheric) to local scale.

## Materials and Methods

In this study we have utilized the following times series over the period January 1970–December 2005, unless otherwise stated. Original sampling frequencies of all selected time series are monthly, unless otherwise stated:

- total copepod abundance (standard units), Gulf of Trieste, Adriatic, eastern Mediterranean
- the northern Adriatic red tides time series (episodes)
- the northern Adriatic mucilage events time series (episodes)
- anchovy stock biomass (standard units), Adriatic (yearly, 1976–2001)
- zooplankton abundance (standard units), Ligurian Sea, western Mediterranean (weekly, November 1966–December 1993)
- SST (°C), Gulf of Trieste, Adriatic, eastern Mediterranean
- SLP (hPa), Gulf of Trieste, Adriatic, eastern Mediterranean
- SST (°C), Ligurian Sea, western Mediterranean
- SLP (hPa), Ligurian Sea, western Mediterranean
- SST (°C), Mediterranean Sea
- SLP (hPa), Mediterranean Sea
- Northern Hemisphere Temperature (NHT) index
- North Atlantic Oscillation (NAO) index

**Figure 1:**
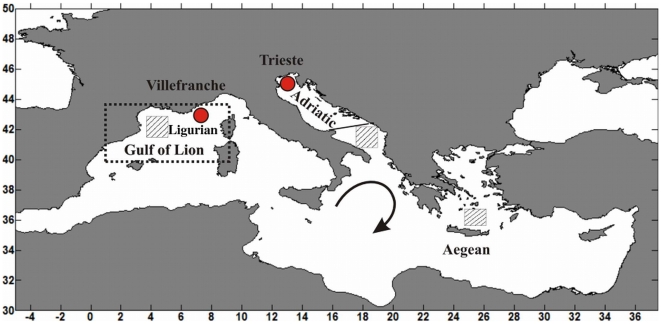
Map of Mediterranean Sea. The red dots show the location of the two long term stations for the collection of zooplankton samples used in this study, in the Gulf of Trieste, Adriatic Sea, eastern Mediterranean, and off Villefranche, in the Ligurian Sea, western Mediterranean. The line at the bottom of the Adriatic indicates the southern limit of the area over which anchovy biomass was estimated. The dotted area in the western Mediterranean represents the area over which monthly SST and SLP data for the north-western Mediterranean were spatially averaged. The curved arrow represents the Ionian gyre reversal of 1987, which lasted approximately until 1997. The striped boxes show approximately the areas of deep water convection in the two sub-basins: these are located in the Gulf of Lyon in the western Mediterranean, while in the eastern Mediterranean, the Adriatic area was dominant until 1987, the Aegean after 1987, for about a decade.

Copepod samples were collected in the Gulf of Trieste, Adriatic Sea (Fig. 1) by vertical hauls from the bottom (18 m) to the surface, with a 200 µm mesh size WP2 net [18]. The data have been collected monthly from April 1970, with a 5 year gap from January 1981 to February 1986, inclusive. The total copepod abundance (number of individuals m^−3^) used for this study was computed as the sum of the abundance of all copepod species per sample.

The Villefranche, Ligurian Sea (Fig. 1) plankton samples used in this study were collected weekly since November 1966 to December 1993. The plankton sampling was performed by vertical hauls from bottom to surface with a Juday-Bogorov net (330 µm mesh size). The zooplankton abundance time series (ind m^−3^) used here is derived from 14 selected zooplankton species encompassing different trophic levels: 5 copepods, 3 chaetognathes and 6 jellies (see [19]).

For this study, the total copepod abundance in the northern Adriatic and the zooplankton abundances in the Ligurian Sea were standardized to zero mean and unit variance, and yearly averaged.

The red tide events affecting the northern Adriatic Sea and the Gulf of Trieste are described by Fonda Umani [20]. For this work we have used presence/absence records starting from 1970.

The northern/central Adriatic mucilage events time series dates back to 1729, and has been reconstructed from scientific papers and several records in old newspapers regarding episodes affecting the north and central Adriatic Sea (see Fonda Umani [21] and references therein). For this work we have used presence/absence records starting from 1970.

The anchovy biomass used in this work was estimated by means of the fisheries stock assessment method Virtual Population Analysis [22], using yearly-averaged catch data relative to the Adriatic area with the Gargano Promontory as the southern boundary (Fig. 1) from 1976 to 2001. In this study the anchovy stock biomass (tonnes) was standardized to zero mean and unit variance.

Monthly sea surface temperature (SST) and sea level pressure (SLP) for the Gulf of Trieste for the period 1970 to 2005 were kindly provided by CNR-ISMAR-Trieste, from their long-term meteorological station located at 45°38′34″N, 13°45′14″E. Temperature and pressure data were averaged over the winter months (Jan-Mar and Dec-Feb, respectively).

Monthly sea surface temperature data for the north-western Mediterranean were downloaded from the iCOADS data set (http://icoads.noaa.gov/), for the period 1970 to 2005. The data were spatially averaged over the area between 39.5–43.5 latitude, and 0.5–9.5 longitude (Fig. 1), and temporally averaged over the winter months (Jan–Mar).

Monthly sea level pressure data of the north-western Mediterranean were downloaded from the NCEP/NCAR Reanalysis data set (http://www.esrl.noaa.gov/psd) for the period 1970 to 2005. The data were spatially averaged over the area between 39.5–43.5 latitude, and 0.5–9.5 longitude (Fig. 1), and temporally averaged over the winter months (Dec–Feb).

The basin (Mediterranean) SST data were downloaded from the iCOADS data set (http://icoads.noaa.gov/), for the period 1970 to 2005. The data were spatially averaged over the entire Mediterranean basin, and temporally averaged over the winter months (Jan–Mar).

The basin (Mediterranean) SLP data were downloaded from the NCEP/NCAR Reanalysis data set, for the period 1970 to 2005. The data were spatially averaged over the entire Mediterranean basin, and temporally averaged over the winter months (Dec–Feb).

The winter Northern Hemisphere Temperature (NHT) data were downloaded from www.cru.uea.ac.uk, HadCRUT3v data set, combined land air and marine surface temperature anomalies on a 5° by 5° grid-box basis, for the period 1970 to 2005. Winter averages cover the months December to February.

The winter North Atlantic Oscillation (NAO) index data were downloaded from www.cpc.ncep.noaa.gov/, for the period 1970 to 2005. Winter averages cover the months December to February.

The normality of all series used was tested using the Anderson-Darling A^2^ test, which is considered one of the most powerful statistics for detecting most departures from normality [23]. In all cases the null hypothesis that the data came from a normally distributed population could not be rejected at alpha level  = 0.05.

The zooplankton series were subdivided in two sub-periods: 1970–1987 (T1) and 1988–2005 (T2) for the Gulf of Trieste series, following Conversi *et al*. [24]; and 1966–1987 (T1) and 1988–1993 (T2) for the Ligurian series, following Molinero *et al*. [19]. The Student's t-test was applied to both series to test the null hypothesis of no difference in the mean abundances over the two periods.

The most commonly investigated regime shift hypothesis is a step change in mean level using parametric or non parametric methods [25]. To identify the period of change in climate proxies, we have utilized the cumulative sum technique, one of the methods based on variations of the mean used for detecting regime shifts [26]. The method consists of plotting the cumulative sum of standardized values over time. Each value of the series is subtracted from a reference value (here the mean of time series), resulting in a new time series of residuals which are used for the calculation of the cumulative sum (S_t_), as follows: each data point y_t_, (t corresponding to time t, from 1 to n) is added to the preceding data point according to the equation: 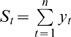. The interpretation is based on the slope of the line on the chart: a constant deviation from the mean of the time series shows a constant slope. Persistent changes from the mean of the time series cause a persistent change of the slope [27].

In addition, to test the regime shift hypothesis as a step change in mean level, we have used a parametric method based on sequential t-test analysis of regime shifts (STARS), developed by Rodionov [28] and modified by Rodionov and Overland [29]. Its advantage is that it provides a probability level for the identified year of regime shift, based on the Student's t-test. The method consists of calculating a Regime Shift Index (RSI), which represents a cumulative sum of normalized anomalies relative to a critical value. In STARS the time scale to be detected is controlled primarily by the cut–off length, which is similar to the cut-off point in low-pass filtering, and determines the minimum length of the regimes for which the magnitude of the shifts remains intact [28], [29]. A longer cut-off length hence identifies the strongest signal (as opposed to many smaller events). In this work we have chosen a large cut–off length (20 years), so to identify just the year of major change in all time series used. We have chosen a probability level equal to 0.01. A tool for the detection of a regime shift based on STARS is available on the website http://www.beringclimate.noaa.gov/regimes [25].

All statistical analyses were performed using MATLAB or ‘R’ software packages.

## Results

### a) Changes in the Adriatic ecosystem, eastern Mediterranean

The Adriatic Sea (Fig. 1) is an elongated basin, stretching southward for 800 Km from the highest Mediterranean latitude (45° 47′) in the Eastern Mediterranean basin. Its main circulation pattern is cyclonic, with the Eastern Adriatic Current flowing northward from the Levantine Basin and the Western Adriatic Current flowing southward along the Italian coast. In the northernmost part of the sea, lays the Gulf of Trieste, a shallow area characterized by highly variable riverine input and strong wind forcing in the winter (Bora). The Gulf of Trieste hosts one of the longest copepod time series in the Mediterranean (1970 - to date, monthly sampling, interrupted Jan-81–Feb 86).

Conversi *et al*. [24], after analyses of the winter SST in the Gulf of Trieste, chose 1987 as boundary year, and showed that the whole copepod community in the area underwent a substantial transformation after that year, which included an approximate doubling in abundance, mostly due to the increase of smaller species (*Oithona*, *Oncaea*), changes in the phenology in the majority of the species, the decline of cold water species (in particular, *Pseudocalanus elongatus*), the appearance of a new species (*Diaxis pigmaea*), and the rise of species found commonly in the southern basin of the Adriatic Sea (i.e., *Paracalanus parvus*). The Ionian gyre reversal of 1987 and consequent augmented input of surface waters of North Atlantic origin in the Adriatic Sea was hypothesized, together with the general warming of the area, as possible explanations for the alteration in the copepod community.

In this work, we have combined total copepod abundance with other biological time series in the area representing additional trophic levels. The results show that the changes after year 1987 encompass more trophic levels and a larger area.

**Figure 2:**
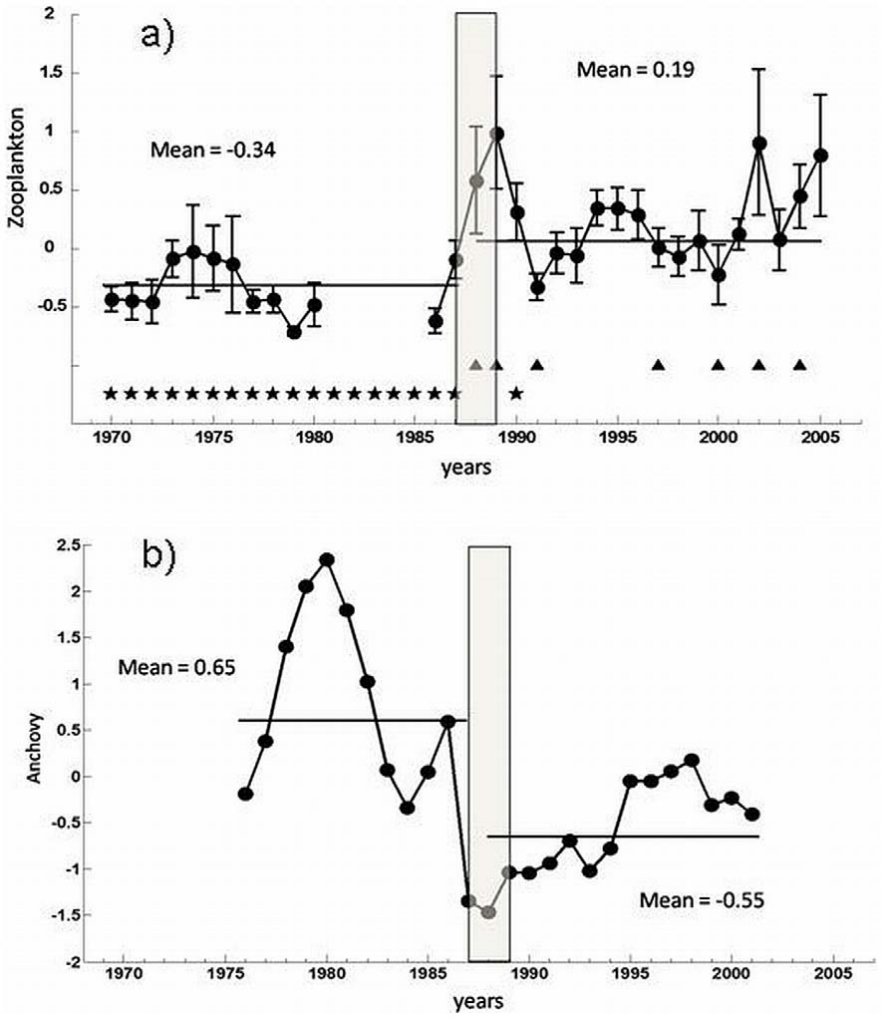
Summary of changes in the Adriatic (eastern Mediterranean) ecosystem at the end of the 1980s. (a): Line: total copepod abundance (standard units) measured in the Gulf of Trieste, 1970–2005. Stars: years in which red tides occurred. Triangles: years in which mucilage events were recorded. (b): anchovy stock biomass (standard units) averaged over the Adriatic Sea. The horizontal lines correspond to the means of the subperiods 1970–1987 and 1988–2005. The vertical grey bar shows indicatively the period of change (late 1980s).

In Fig. 2a we show standardized total copepod abundance in the Gulf of Trieste, red tide occurrences, and mucilage events in the northern Adriatic, for the period 1970–2005. When the standardized copepod time series is divided in two periods, 1970–1987 (mean = −0.34, st.dev = 0.22) and 1988–2005, a large increase in total copepod abundance can be seen in the second period (mean = 0.19, st.dev = 0.37), during which the mean is 0.53 standard units larger than during the first period. A Student t-test was applied to the two sub-series, and the null hypothesis of no change in the abundances between periods was rejected with p<0.01.

We note however that the change encompasses additional trophic levels. In fact, the chain of red tides – mucilage events in the northern and central Adriatic Sea is centered around 1987 as well (Fig. 2a).

Red tides in the Northern Adriatic are mostly due to dinoflagellate blooms that can cover large areas and can be potentially toxic (e.g. *Prorocentrum lima*, *Lingulodinium* (*Gonyaulax*) *polyedrum*). At times red tides affected the whole Northern Adriatic, as in September – October of 1984 [30], but they were more frequent in confined areas, especially in a narrow coastal belt along the western coast, where the frontal system persists even during summer months [20]. Red tides events started in 1968 and affected the northern Adriatic each year, until 1987 (Fig. 2a). After the reappearing of mucilage in 1988, there was another red tide season only in 1990 [31].

Mucilage events are huge accumulation of dissolved (colloidal) organic matter that can cover the sea surface for hundreds of square kilometers and underwater can assume the aspect of large clouds up to 6–7 m long and >2 m large [32]. They can foul fishing nets and inundate benthic communities, with heavy repercussions on fishing and tourism industries. In a recent paper, Danovaro et al. [33] pointed out also to the potential spreading of microbial pathogens via mucilage transfer. Mucilage events tend to happen in summer, after the demise of the spring diatom bloom. The cause of these events is still under debate, but recently it was demonstrated to be mostly due to the uncoupling between primary production and bacterial consumption [34]. The northern Adriatic mucilage events time series, the longest series in the Adriatic, dates back to 1729. After 1729, mucilage events have been noted in 1872, 1880, 1903, 1905, 1920, 1930, 1949, possibly 1951, and, after disappearing for almost 40 years, they reappeared in 1988 and continued in 1989, 1991, 1997, 2000, 2002, 2004 and 2007 [21], [35], [36], [33]. The events recorded for the period 1970–2005 are reported in fig. 2a. What is most relevant with respect to our study is their absence during the first period of the series, and their recurrent appearance since 1988.

In synthesis, there was a switch in the occurrences of red tides and mucilage, centred around year 1987, with red tides affecting the area yearly from 1968 to 1987, and mucilage, not present since 1951, reappearing in 1988 and occurring repeatedly since. The explanations for this alternance are still under debate. Degobbis *et al*. [35], find qualitative changes in the phytoplankton community (increased diatom contribution, decreased diversity, different dominant species) in the 1980s *vs*. the 1970, and propose that climate changes in the 1980s and subsequent changes in the hydrological regimes might be the cause. We propose that such changes are additionally linked to the changes in the Mediterranean circulation at the end of the 1980s, in particular to the changes in salinity and nutrient budgets due to the modified relative contributions of waters of North Atlantic and Levantine origin in the Adriatic Sea caused by the Ionian reversal of 1987 (see Section c). Such alteration might have affected the phytoplankton community structure, as well as the coupling of primary production and bacterial consumption.

The year 1987 seems of importance also for higher trophic levels in the Adriatic Sea. Of particular relevance are the long term changes in the anchovy (*Engraulis encrasicolus* L.) stock biomass, one of the most important commercial species of the northern and central Adriatic. In Fig. 2b we show the time series of anchovy biomass, estimated by Virtual Population Analysis (see [22]), over the Adriatic area. It can be seen that the population declined since the late 1970, reaching a minimum in 1987. The explanations for such abrupt reduction so far include climate forcing, i.e., a positive NAO index and lower autumn SSE-ESE quadrant wind stress, autumn flow rate of the Po river [22]; modified inflow of Mediterranean waters in the Adriatic Sea (and associated salinity changes) due to modified pressure differences between the mid north Atlantic and the southeast Mediterranean [37]; overfishing [38]; anchovy mortality due to increased predation of eggs and larvae by the jellyfish *Pelagia noctiluca* in the years from 1977 to 1985 [39]; a combination of the above [40]; and the mucilage presence [39], although we note that the latter began after the anchovy's collapse. As Grbec *et al*. [37] point out, landings of different species changed synchronously in all ports around Italy and eastern Adriatic, so that local fishing effort could not explain alone these synchronicities. We observe that the year 1987 does not signify a step change in the anchovy biomass, as was the case for zooplankton abundance, but instead marks the end of a decade-long decline. We also note that while anchovy biomass decreased all over the Adriatic Sea, copepod abundance (data limited to the Gulf of Trieste) increased in the second period, which can suggest a top-down trophic control.

Overall, we observe that all our biological indicators for the Adriatic Sea point out to a period of change in the late 1980s.

### b) Changes in the Ligurian ecosystem, western Mediterranean

In the western Mediterranean basin, the Ligurian Sea (Fig. 1) is one of the most productive zones. Hydrography of the area is governed by a permanent cyclonic circulation, the Northern current, shaped by the flux of water masses through the Corsica Channel [41]. Previous analyses of the Villefranche plankton records identified synchronous interannual variations in the North Atlantic climate and the north-western Mediterranean pelagic ecosystem, in particular anomalous events, i.e. outbursts and drops in the abundance of copepods, chaetognaths, doliolids, salps and jellyfish [42], [19].

**Figure 3:**
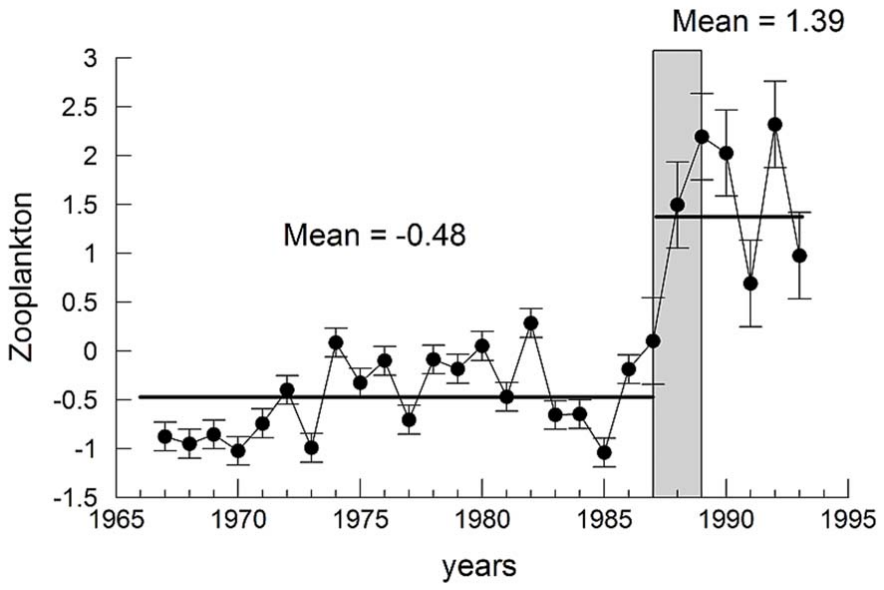
Changes in zooplankton abundance (standard units), measured off Villefranche, Ligurian Sea, western Mediterranean, 1966–1993. The horizontal lines correspond to the means of the subperiods 1966–1987 and 1988–1993. The vertical grey bar shows indicatively the period of change (late 1980s).

In Fig. 3 we show the zooplankton time series collected in the Ligurian Sea over 1966–1993. When the series is divided in two periods (1966–1987 and 1988–1993), we find that also in this area zooplankton abundance increases in the second period, during which the mean is 1.87 standard units larger than the first period mean. A Student t-test was applied to the two sub-series, and the null hypothesis of no change in the abundances between periods was rejected with p<0.01.

The increase in period 2 appears to be mainly due to the gelatinous component of the series. It is possible that, during the second period, warmer temperatures and lower water column mixing resulted favourable for gelatinous zooplankton, both herbivorous and carnivorous - i.e. doliolids, salps and jellyfish and to some copepod species (i.e. *Centropages typicus* and *Acartia clausi*), and unfavourable for chaetognathes and summer-autumn copepods, which decreased.

Other authors have found additional environmental changes in this basin around the same period. Goffart *et al*. [43] analyzed several years of phytoplankton collection during the period 1979–1998 off Corsica, in southern Ligurian Sea. They found a drastic reduction in phytoplankton biomass during the spring bloom, accompanied by a modification in its structure from diatom-dominated, up to 1986, to non-siliceous phytoplankton afterwards, which they attributed to decreasing Si availability after 1986. They also found unprecedented, system-wide changes in pelagic and benthic primary producers, benthic fauna and fish assemblages between 1986 and 1988.

Because these are different data collections in far away sites, one cannot to state whether the observed changes are associated across sites, and whether there is a causal relationship between the opposite signs of phytoplankton and zooplankton variations: however, spatio-temporal studies in other marine systems suggest that environmental forcing often prevails over trophic pressure (see [44], on the North Sea). These results however highlight that also in the western Mediterranean, independent studies with distinct data sets indicate that several compartments of the pelagic ecosystem underwent a period of change in the late 1980s. While the hypotheses to explain it at regional scale are still underway [43], [45], in this work we propose that the changes observed in the Adriatic and Ligurian seas are an ecosystem-wide response to some type of large scale forcing.

### c) The physical system: hydrographic and circulation changes in the Mediterranean Sea

A review of the Mediterranean physical oceanography literature provides evidence to support this argument, by indicating the existence of peculiar conditions at the end of the 1980s.

In fact, 1987 appears to be a year of change for the entire basin surface circulation. Demirov and Pinardi's [46] simulations of the interannual surface Mediterranean circulation from 1979 to 1993 identify two periods, 1981–87 and 1988–93, which differ in precipitation and winter wind regimes.

Pinardi *et al.* [47] and Korres *et al.* [48], using data-validated simulations describe the dramatic reversal of the Ionian gyre in the summer of 1987 from its “usual” cyclonic state to an anticyclonic pattern (a simple graphic representation is reported in Fig. 1). In particular, they show a reversal in the surface current directions in the Ionian Sea, with the Atlantic/Ionian stream (and associated nutrients and hydrographic properties), branching further northward, at 35.5°N, and link it to the surface circulation changes to the previous winter anomalies in the winds and heat fluxes. The alteration lasted approximately 10 years, until 1997, when the gyre re-reversed, as shown by altimetry data [49] and models [50], [51], [52].

However, the largest modification in the Mediterranean circulation observed in the last decades involved the shift in deep water formation in the Eastern basin from its usual source in the southern Adriatic to a new source in the Aegean Sea (see Fig. 1), and started also at the end of the 1980s. This phenomenon, called the Eastern Mediterranean Transient (EMT), started after 1987, peaked approximately between 1989 and 1995 and started relaxing at the end the 1990s [53], [54], [55], [56], [57]. It was accompanied by a vast rearrangement of the entire water column circulation and associated chemico-physical properties. Several hypotheses have been put forward to explain the causes of this phenomenon, which include the exceptionally cold winter of 1987 and the Ionian gyre reversal in the summer of 1987 [55], which disrupted the flow of the North Atlantic Water from the Ionian into the Levantine Basin causing a large net increase of salt in the global budget of the Eastern Mediterranean [58]. The gyre re-reversed in 1997 [49], probably in relation with the relaxation of the Eastern Mediterranean Transient [59]. Overall, the EMT affected the thermohaline and bio-chemical properties of the Mediterranean waters, leading to substantial modifications in the water exchanges between the western and eastern Mediterranean, and the Adriatic and Aegean Seas [60], [37].

Observed data from the MEDAR climatology also point to a Mediterranean-encompassing change in the late 1980s: a closer look at the temperature and salinity surface-to-bottom time series for 1950–2000 evidences a clear change in the late 1980 (Fig. 1 and Fig. 2 in [61].

We note here that several independent studies, not focusing on regime shifts (hence, unbiased), involving different techniques - from time series analyses, to *in situ* observations, and to general circulation models – all point to the late 1980s as a period of major change in the Mediterranean hydrography, surface circulation, and deep convection. We therefore hypothesize that such changes, and associated modifications in water properties, underlie, together with the temperature changes, the ecological alterations described in both sub-basins. We further hypothesize the presence of a larger scale alteration around this period, which is beyond all phenomena described.

### d) From northern hemisphere to local scale

In order to verify this idea, we have hypothesized that the local marine system is linked (i.e., shows similar patterns) to the regional and basin physical system, and to the larger scale climate (as indicated by north hemisphere indices).

For our analyses we have chosen, as large scale climate indices, the winter North Atlantic Oscillation (NAO) index, because the NAO it is the dominant winter mode in the Atlantic area [62], with effects reaching into the Mediterranean Sea [63]. In addition, we have selected the winter Northern Hemisphere Temperature (NHT), an index that combines land air and marine surface temperatures in the northern hemisphere, because temperature seems to play a critical role in triggering ecological regime shifts [64].

As Mediterranean basin hydrological proxies we have chosen the winter sea level pressure (SLP) and sea surface temperature (SST) because the Mediterranean circulation is mostly forced by the winter climate [48].

We have chosen as proxies for the regional/local climate the winter SLP and SST in the Ligurian Sea (box in Fig. 1), western Mediterranean, and in the Gulf of Trieste, Adriatic Sea, Eastern Mediterranean basin.

As local biotic proxies we have chosen the total copepod abundance in the Gulf of Trieste, and zooplankton abundance in the Ligurian Sea, because they have shown a rather abrupt shift at the end of the 1980s [19], [24].

**Figure 4:**
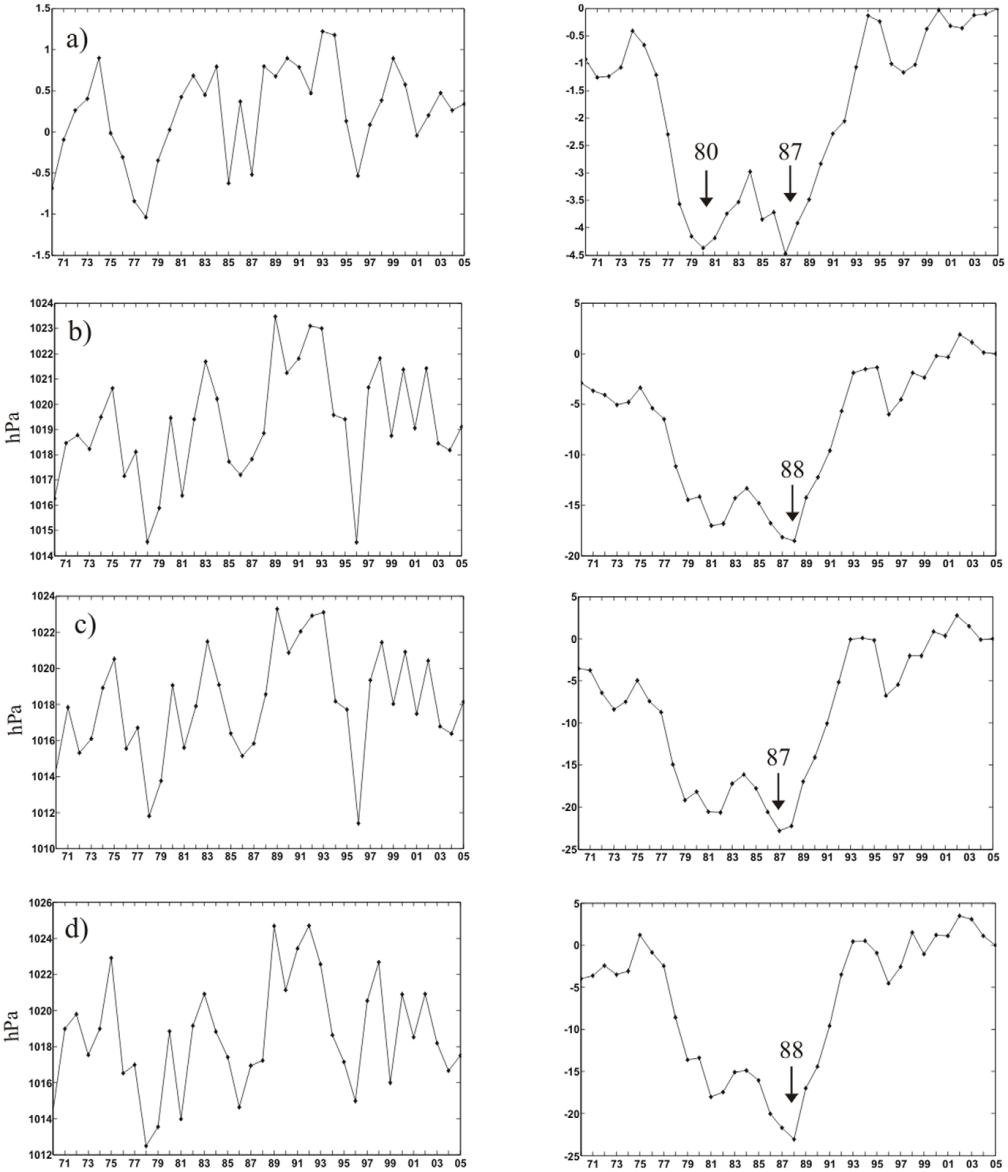
Time Series (left panel), and Cumulative Sums (right panel) of pressure based variables, over 1970–2005. a) winter North Atlantic Oscillation (NAO) index; b) winter average SLP over the Mediterranean basin; c) winter average SLP over the north-western Mediterranean; d) winter SLP in the Gulf of Trieste. In the cumulative sum panels the year of change is indicated by an arrow.

**Figure 5:**
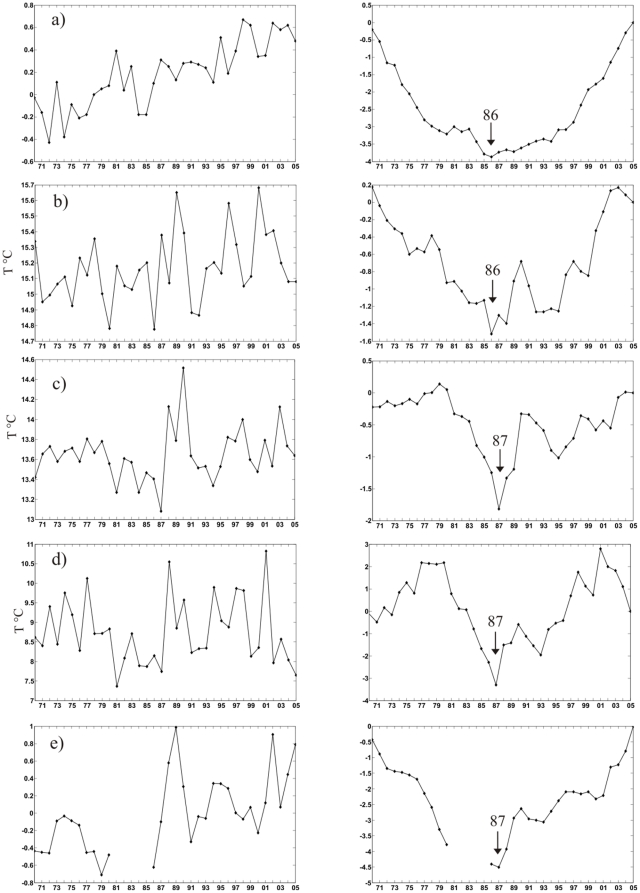
Time Series (left panel), and Cumulative Sums (right panel) of temperature based variables, over 1970–2005. a) winter Northern Hemisphere Temperature (NHT) index; b) winter average SST over the Mediterranean basin; c) winter average SST over the north-western Mediterranean; d) winter SST in the Gulf of Trieste; and e) total copepod abundance in the Gulf of Trieste (standard units). In the cumulative sum panels the year of change is indicated by an arrow.

The time series of these variables for the period 1970–2005 are shown in Fig. 4 and 5 (left panels). In Fig. 4a it can be seen that the NAO index remains in positive phase since 1988 (with the exception of 1996). In the Mediterranean, the winter SLP at basin and sub-basin scales presents a period of high pressure at the end of the 1980s–early 1990s (Fig. 4b–d).

Starting in 1986 and continuing, the winter NHT anomalies are always positive (Fig. 5a). The winter Mediterranean SST (fig. 5b) shows higher variations after 1987 and an overall increase through time. The winter SST in the Ligurian Sea (Fig. 5c) behaves similarly, with a marked increase after 1987. The Gulf of Trieste winter SST (Fig. 5d) shows higher variability after 1987 and no overall trend. Total copepod abundance in the Gulf of Trieste shows a drastic increase around the end of the 1980s (Fig. 5e).

The right panels in Fig. 4 and 5 show the corresponding cumulative sums. Cumulative sum is a method used to detect regime shifts which is based on differences of the means [26]. As it can be seen in Fig. 4a, the method identifies 1987 as year of change for the winter NAO index (in addition to 1980). The Mediterranean winter SLP shows a similar pattern, with the year 1987 or 1988 (in addition to 1981) identified as year of change at all investigated scales (Fig. 4b–d). With regard to temperature, 1986 is identified as year of change for the winter NHT (Fig. 5a), for the winter Mediterranean SST (Fig. 5b), and for Ligurian zooplankton (not shown); and the year 1987 is identified as year of change for winter SST at sub-basin scales (Fig 5c–d) and for total copepods in the Gulf of Trieste (Fig. 5e).

In other words, there is a quasi-concurrent, abrupt period of change for all physical variables (SLP and SST) investigated at all scales - from the local (Trieste), regional (Ligurian), basin (Mediterranean), to the hemispheric scale (NAO and NHT indices), as well as for the local biological indicators – which occurs within a very short period, around 1986–1988.

**Figure 6:**
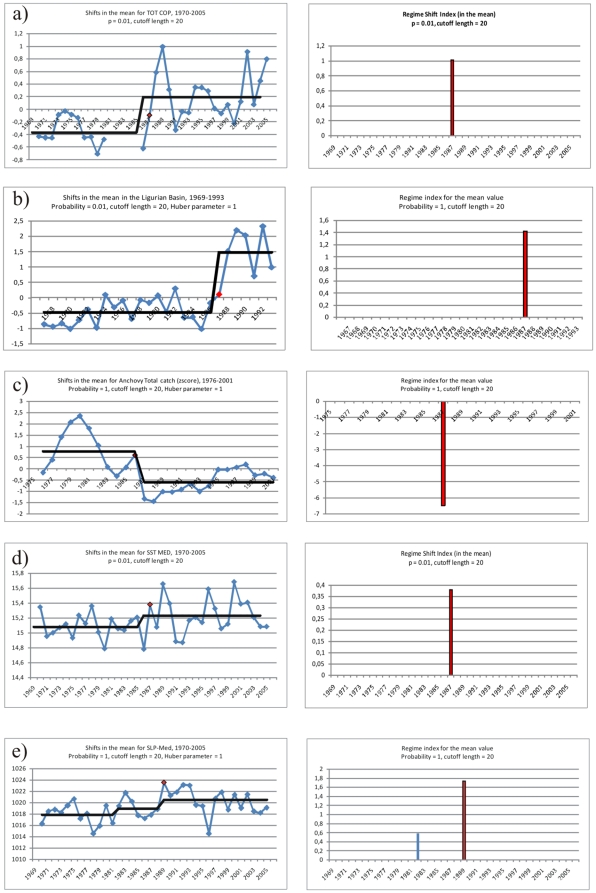
Regime shift detection (left) and Regime Shift Index (right). a) total copepods of the Gulf of Trieste, b) zooplankton in the Ligurian Sea, c) anchovy catch in the Adriatic, d) winter Mediterranean SST, and e) winter Mediterranean SLP. In the left panels the blue lines represent the time series, the black lines the stepwise trend showing the regime shift in the mean detected by STARS method, the red dots the year identified with the STARS method with p≤0.01. In the right panels, the red bars represent the Regime Shift Index (RSI) identified with the STARS method.

To further the hypothesis of a regime shift in the Mediterranean Sea (and beyond) we have then utilized the Sequential T-test Analysis of Regime Shifts (STARS) described by Rodionov and Overland [29], which has the advantage to provide a confidence level for the years detected. In Fig. 6 we show the results of the testing for several biological and physical properties: total copepods in the Gulf of Trieste, mesozooplankton in the Ligurian Sea (local biological response), anchovy catch in the Adriatic (higher trophic level, regional biological response), and Mediterranean winter SST and SLP (basin-wide physical proxies). The time series and step trends are shown in the left panels. In the right panels are reported the respective Regime Shift Indices (RSI). They are all statistically significant with probability p≤0.01.

Also this technique identifies a step-change in the late 1980s in the variables. These results are similar, but not identical, to what seen with the cum sums method, which is reasonable because the methods are different. In particular, STARS identifies the year 1989 as year of shift in the Mediterranean SLP (cumsums indicated that SLP Med started changing after 1988), which again is after the year of temperature change at Mediterranean scale, 1987 (with cumsums, the years of change of temperature were 1986–87, depending on basin scale). The year identified for all three biological indicators is 1987, although the sign is different for the higher trophic level indicator (anchovy catch), which had undergone a long decline since the late 1970s. This suggests that Mediterranean SLP is not a good predictor for biota changes. A causal link between climate indicators and biological variability, if any, is more likely related to temperature and/or circulation changes. A climate-biota transfer mechanism is proposed in the session *Climate control*.

Although co-occurring changes do not demonstrate a causal link between large and local scale properties, or between physical and biological indicators, the purpose of this article is to point out the existence of a quasi (meaning within a few years) synchronous co-variation properties at all scales investigated. This is the first necessary step towards establishing that the different scales are connected, possibly as part of a larger scale variation, and eventual causal relationships. Additional work, involving several basins and properties may confirm this line of thought.

All above results, and the additional information found in the literature, carry several implications: a) a major change is shown by physical and biological properties of the Mediterranean Sea in the late 1980s, therefore identifying a regime shift in this sea; b) the alterations are multi-scale, i.e. happen quasi-synchronously in the local hydrography (as represented by Gulf of Trieste winter SST and SLP), in the regional hydrography (as represented by north-western Mediterranean SST and SLP), in the basin hydrography (as represented by Mediterranean SST and SLP), and in the northern hemisphere climate (as represented by the Northern Hemisphere Temperature and the NAO index); and c) this leads to hypothesize that the phenomenon underlying the Mediterranean regime shift is larger than the Mediterranean basin scale.

## Discussion

### The larger scale perspective: the North, Baltic, and Black Seas regime shifts

The several changes that happened around the end of the 1980s in the Mediterranean Sea, involving the physical and biological systems, appear indeed to be another tile in a larger scale puzzle.

In fact, in the same period, regime shifts have been reported in several basins around Europe. In the North Sea, in the late 1980s a stepwise change in regional (wind and sea temperature), and large scale properties (NHT), was identified as a regime shift. The phenomenon affected all trophic levels, and was concurrent to a substantial increase in the winter-months oceanic inflow from the Atlantic into the northern North Sea, which was in turn associated to the NAO positive phase [65], [66]. Chlorophyll-*a* increased even as nutrients decreased, and was in fact correlated to North Sea SST, transparency and inflow [67], suggesting that phytoplankton biomass is driven more by climate and water circulation than by food availability (as indicated by nutrient concentration). At the same time zooplankton abundance increased, as well as horse mackerel catch, while salmon catch and gadoid recruitment decreased [65], [44], [66], and abrupt shifts were found even in the benthic realm [68]. A closer look at the calanoid copepod assemblages showed the entire community undergoing a reorganization of the biogeography, with a northward extension of the warmer species of more that 10° latitude and a decrease in the number of colder water species [10]. The above changes were associated to variations in the pressure distribution over the Atlantic, as shown by the NAO index, and to changes in the sea temperature, as indicated by the Northern Hemisphere Temperature (NHT) [44], [64], and the Atlantic Multidecadal Oscillation (AMO), a large scale sea temperature signal of about 60 to 70 years period, with a considerably larger spatial extent than the NAO [10]. Of particular interest are Beaugrand's *et al*. [64] findings that marine ecosystems are not equally sensitive to climate change and reveal a critical thermal boundary, around 10°C, where a small increase in temperature triggers abrupt ecosystem shifts across multiple trophic levels.

A regime shift was also identified in the nearby Dutch Wadden Sea [69], which was attributed to temperature and weather conditions.

In the Baltic Sea, Alheit *et al*. [15] split the temperature SST series in two periods, 1970–1987, and 1988–2003, and found an average increase in Spring, Summer and Autumn SST by 1–1.5°C, which they linked to the heat fluxes associated with the positive phase of the NAO, and with inflow from the North Sea, while Möllmann *et al*. [70] consider both atmospheric and anthropogenic pressure. Changes in the planktonic community included a sharp increase in spring phytoplankton starting in 1987–1988 [71], and the strong decline of *Pseudocalanus elongatus*, which started in the late 1980s–early 1990s [72]. *P. elongatus* was replaced in its role as dominant copepod species by *Temora longicornis*, and concurrently the fish community shifted from cod to sprat [70]. Alheit *et al*. [15] also noted the synchronous regime shifts happening in the North and Baltic Seas, and their similarity, and considered this as a response to the NAO.

In the late 1980s the Black Sea ecosystem shifted from a small-planktivore-fish (anchovy) controlled system to a gelatinous-carnivore controlled system. The small-planktivorous fisheries collapsed and the invasive carnivore-ctenophore *Mnemiopsis leidyi* reached enormous biomass levels [73], [74]. Two main hypotheses were proposed to explain this shift: heavy fishery exploitation and subsequent trophic cascade [75], [76], and the invasion of the alien ctenophore (eased by an eutrophication-stressed system) which fed on the food supply of the anchovy, as well as on its eggs and larvae [73], *M. leidyi*'s invasion was attributed to introduction via ballast waters from ships coming from the USA in the early 1980s, although it was noted that the ships were coming also prior the 1980s, and thus some other environmental factor must have triggered the outburst [77]. The possible role of climate on the outburst of *M. leidyi* and the decline of the anchovy stocks so far attracted less attention: Niermann *et al*. [78], drew attention to changes in the wind stress, mixing and stratification, and Oguz *et al*. [79] found that variations in nutrient concentrations and phytoplankton biomass appeared to be governed by the North Atlantic Oscillation and the East Atlantic-West Russia patterns. Recently, Oguz *et al*. [80] focused on the relative contributions of these hypotheses and of climate on causing the switch, through a bidrectionally coupled model, and concluded that neither hypothesized mechanism alone (overfishing, or ctenophore impact) would explain it, and that such switch could not happen without concurrent extremely strong environmental perturbations.

While the described shifts are rather different in their manifestations, they all have in common the fact that they happened about synchronously, during a short period of few years, 1986–1989. In this work we propose that this can hardly be considered a coincidence, and that they all participate in a larger scale phenomenon.

### Analogies across basins

By looking more in depth at the reported phenomena we can find that some patterns of the Mediterranean and the other European seas shifts appear strikingly similar in spite of the geographical distance and the vast difference in basins and ecosystems, and again have been found by independent studies, mostly not related to regime shifts, via different analytical methods.

Besides the common period of divide (late 1980s), for example, we find that the general SST warming is not equally distributed in all seasons: for instance, in the Baltic and in the north Adriatic Seas, Spring, Summer and Autumn SST increase by 1–1.5 C, while there is no trend in the Winter SST [15], [24].

The direct role of temperature on copepod abundance has been studied by several authors [81], [64], and even small changes in the season of maximum reproduction, may result in significant changes in the species abundance and distribution [82]. Phenological changes associated to changes in temperature seem to be particularly relevant also in the late 1980s: shifts in the phenology of several zooplankton taxa, starting in the late 1980s, are reported in the Gulf of Trieste, Mediterranean Sea [24]. In the central North Sea, Edwards and Richardson [83] have associated changes in Spring SST to shifts in the phenology of functional groups across all trophic levels in the marine pelagic community; a closer look at their results (their Fig. 2) reveals that the timing of the seasonal cycle for each functional group changed abruptly in the late 1980s.

Beaugrand *et al*. [64] suggest the existence of a critical thermal boundary, around 10°C, where a small increase in temperature triggers abrupt ecosystem shifts seen across multiple trophic levels. The independent work by Oguz *et al*. [80] finds a thermal threshold for high *Mnemiopsis* production in the Black Sea, which happens to be the same value (10°C).

Another example of striking similarity in the ecological response in far away basins is the dramatic decline shown after 1987 by *Pseudocalanus elongatus* both in the Baltic Sea, where it was the dominant species before 1987 [72], and in the Gulf of Trieste, where exists a relict artic population found only there, which underwent a 75% reduction since 1987. The increase in summer SST might explain the decline of this cold-water, summer-peaking species [24].

A further example of analogies is given by the increase in the carnivorous gelatinous component of plankton at the end of the 1980s in both the Mediterranean (Ligurian and Adriatic seas) and the Black seas [39], [45], [80]. In the Ligurian Sea the increase was attributed to climate-induced higher temperatures and low water column mixing. In the Black Sea the outburst of the exotic ctenophore *M. leidyi* was attributed to eutrophication/fishing/climate. We observe here the again remarkable coincidence in the timing of the increase of the carnivorous gelatinous components in basins far away, and suggest that their role in shaping the food chain is more important than previously thought. A comparative analysis of these ecosystems, might elucidate the relative role of the large scale (climate) *vs*. the local scale (fishing, eutrophication) drivers on this component of plankton.

The anchovy fishery collapsed at the end of the 1980s in both the Adriatic Sea and in the Black Sea [22], [74]. In both systems different, often contrasting hypotheses were made. They included climate forcing, overfishing and competition/predation by gelatinous plankton (the jellyfish *Pelagia noctiluca* in the Adriatic Sea, the ctenophore *Mnemiopsis leidyi* in the Black Sea). In the Adriatic Sea Grbec *et al*. [37] observed that landings of different species changed synchronously in all ports around Italy and eastern Adriatic, so that local fishing effort could not explain alone these synchronicities. In the Black Sea the model by Oguz *et al*. [80] indicates that neither overfishing or ctenophore impact alone could cause the observed collapse in fishery in the Black Sea, and that environmental forcing was necessary. In both seas an extreme event of cooling took place in 1987 [55], [78], which further suggests that climate-induced circulation changes may have played a significant role in shaping the local food webs in both basins. We note that, owing to the life history traits of anchovy, i.e. a short life cycle and a diet dominated by pelagic copepods, and to its vulnerability in the early stages to gelatinous plankton predators, its recruitment appears particularly sensitive to changes in environmental forcing, hence, it is plausible that its long term variations constitute an ecological proxy of pelagic environmental changes. A joint analysis of these two systems may provide answers that cannot be otherwise found.

These examples are by no mean exhaustive, but provide indications that synchronicities exist not just in the timing of the regime shifts, but also between ecosystem components in unconnected basins. Future work should further develop the links (if any) among systems, through comparative analyses focusing on large-scale climate patterns *vs*. local (e.g., eutrophication) mechanisms, involving multiple basins and ecosystems.

### Climate control

In this section we summarize what has been proposed with regard to the link between climate and the reported regime shifts, with the warning that is a very fast evolving field.

In the North and Baltic seas the cause of the late 1980s regime shift was partly associated to a switch in the behavior of the winter North Atlantic Oscillation (NAO) from a negative phase to its longest ever positive phase, and associated increased sea surface temperature, strong westerly winds [66], [15], [69], and increased inflow of warm, salty water from the Atlantic Ocean [84], [85], [66].

In the Black Sea, Oguz *et al*. [79] found that atmospheric processes over the North Atlantic and Eurasia (the North Atlantic Oscillation and the East Atlantic-West Russia) were responsible for large part of the interannual variations of the pelagic food web, complementing the top-down and bottom-up anthropogenic effects.

The Mediterranean Sea has also been associated to changes in the northern hemisphere climate. The two different periods in the Mediterranean surface forcing and especially their abruptness are explained by Demirov and Pinardi [46] with phase matching of long-term variations in NAO (in particular its intensification at the end of the 1980s), the Pacific–North American Pattern, and the Eurasian Pattern. In the western Mediterranean, the NAO is associated to the water mass transport through the Corsica channel [86] and to the western Mediterranean deep water formation in the Gulf of Lyon [61]. In the Adriatic Sea, Grbec *et al*., [37] find that the low frequency variability of salinity and the inflow of water masses from the Aegean Sea are related to variations in the pressure relative to the sector North Atlantic – central Mediterranean. Significant links have been also found between the North Atlantic Ocean climate and temperature in the Adriatic and the Aegean in the upper layer (0–200 m) [87], as well as with the sea surface temperature in the Eastern Mediterranean (see [88] and references therein), although the sign of the relationships is opposite to that found in the Western Mediterranean.

Most of the studies focusing on climate impacts on the different marine ecosystems have so far involved the NAO, in part because the plethora of literature showing the magnitude of its effects and their extensions to a number of ecosystems [7], and in part for ease of study. However, temperature itself might play an even more important role for pelagic distribution, abundance, and phenology, as suggested by the recent works by Edwards and Richardson [83], Kirby *et al*. [89], Oguz *et al*. [80], Beaugrand *et al*. [64], [10], Koeller *et al.* [90], Greene *et al*. [82], and Ottersen *et al*. [91]. Of particular relevance is the identification, for the North Sea, of a thermal threshold around 9–10°C, and its likely crucial role in triggering an ecological regime shift [64].

Although temperature warming and cycles (e.g., NHT, AMO), and changes in the large scale pressure patterns (e.g. NAO and other climate indices) are both manifestations of climate, the mechanisms of transmission to the ecosystem are different.

**Figure 7:**
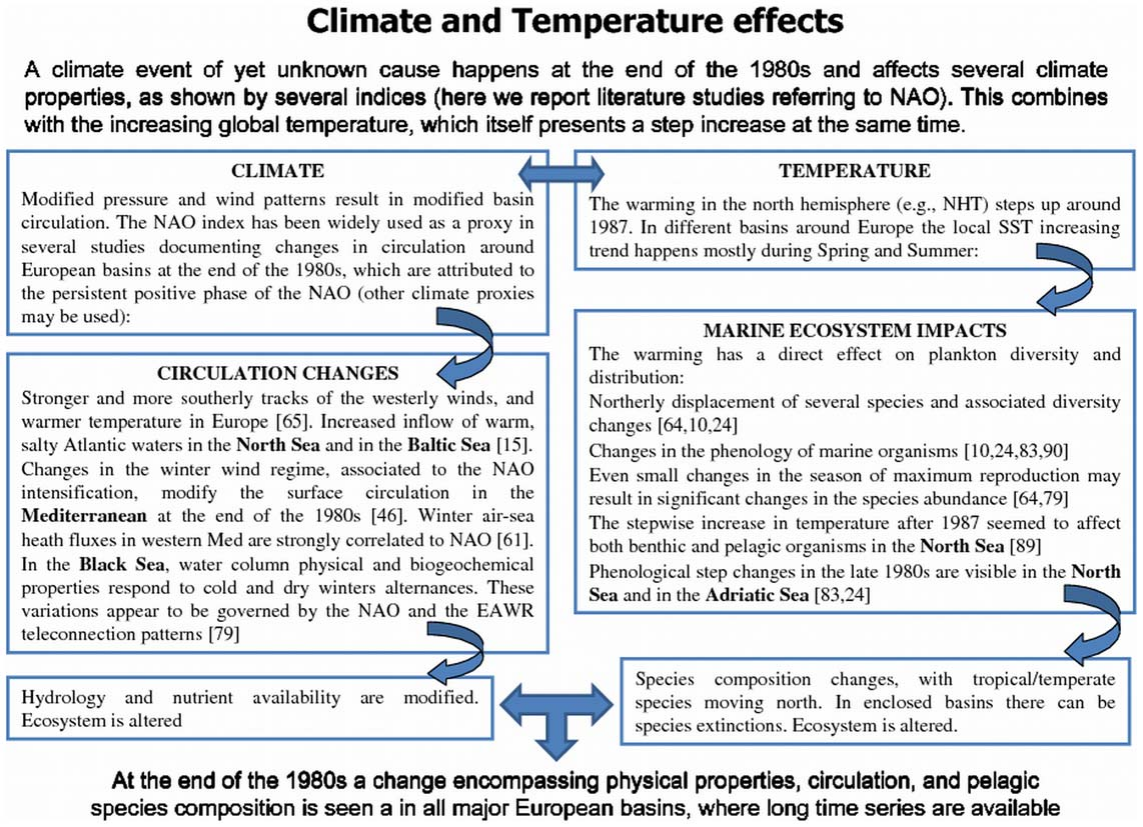
Simplified scheme showing different impacts of atmospheric patterns (as exemplified by the NAO index) and global temperature (as exemplified by the NHT index) on marine ecosystems. The numbers in square brackets indicate supporting references.

We now propose our hypotheses regarding different means of climate transmission from large (hemispheric) to local (basin) scale, and from the physical to the biological realm, via a simplified scheme, shown in Fig. 7. In synthesis:

1. The northern hemisphere warming trend (as exemplified by the NHT index) exhibits an abrupt increase starting circa 1986–87.
2. Also the northern hemisphere climate changes abruptly in the second half of the 1980s. This event of yet unknown cause/origin affects several atmospheric properties, such as sea level pressure and wind regime patterns (as exemplified by the NAO index, but not limited to it).
3. The above changes shape, through different mechanisms, yet synergistically, the local pelagic communities:
4. Changes in the pressure and wind patterns modify inflow, circulation, mixing and stratification in the European basins, with subsequent changes in hydrology and in nutrient availability. Several studies point to this connection, among which Reid *et al*. [65]; Alheit *et al*. [15]; Rixen *et al*. [61]; Oguz *et al*. [79].
5. The warming displaces warmer species northward and affects species distribution and local diversity. Phenological traits, such as the period of maximum abundance, are modified by the warming, especially during critical reproductive periods. Several very recent studies point out to the role of temperature, among which Edwards and Richardson [83], Kirby *et al*. [89], Oguz *et al*. [80], Beaugrand *et al*. [64], [10], Koeller *et al.* [90], Greene *et al*. [82], and Ottersen *et al*. [91].

With regard to the Mediterranean Sea, our review of the physical circulation literature points out to a major change in surface and deep circulation in the late 1980s, diversely attributed to various climate factors. Our analyses of several biological and physical properties at various scales, also identify a substantial change in the late 1980s.

We hypothesize that the climate-biota transmission mechanism is the following: the late 1980s changes in the Mediterranean circulation are due to an alteration in the northern hemisphere climate, which is the same alteration underlying the other reported European shifts, whose signature is seen in the NAO and in the NHT, and which we believe is likely to be found in other climate patterns. The changes in the overall Mediterranean circulation result in modifications in the relative proportion of different water masses in the sub-basins, with corresponding alterations in their hydrographic and nutritive properties (e.g, the increased contribution of waters of North Atlantic origin in the Adriatic Sea). These changes in the water properties combine with the concurrent increase in the temperature, and affect the pelagic community, with resulting shifts in species phenology, reductions and increases in abundance of distinct species, shifts in dominant species, and shifts in the community structure. Whether such modifications can be associated to a particular thermal threshold in the Mediterranean Sea, as seen in the North Sea by Beaugrand *et al*. [64] is too early to say, but worth investigating.

### Conclusions and perspectives

Both our analyses and our review of various independent, multidecadal studies in the Mediterranean Sea - using distinct data and different analytical tools (retrospective analyses, oceanographic *in situ* measurements, general circulation models) - all point to an abrupt change, identifiable at the end of the 1980s, involving both the physical and the biological systems, which can be considered a regime shift.

This regime shift affected the pelagic community (as indicated by plankton, jellies, fish, mucilage, red tides, anchovies) in the western and eastern Mediterranean basins. It also involved the Mediterranean SST, SLP, surface circulation, and we suggest that the deep water convection shift that started in the eastern Mediterranean after 1987 (the East Mediterranean Transient) is also part of the Mediterranean physical regime shift, which is the first interpretation of the kind.

We suggest that the combination of circulation alteration and temperature increase affected the pelagic communities in the Mediterranean sub-basins. In this framework, it is worth noting that findings for the atmosphere at European [88] and Mediterranean scale have shown the last decade as probably the warmest during the last 50 years for whole Mediterranean (>95% confidence level [61]). This probably means that a return to the environmental regime previous to the shift appears low, and therefore bears implications in the developing of management policies.

The results presented here are of ecological relevance as the Mediterranean Sea supports ∼10% of the world marine diversity [92], plays a key role as heat reservoir and source of moisture for surrounding land areas [93], and further emerges as one of the most prominent climate change hotspots [94].

As a final point, in the same late 1980s period, regime shifts are reported in the North Sea, the Baltic Sea, and the Black Sea. The quasi-synchronous occurrences of regime shifts in widely separated marine systems may be explained in two ways: a) occurring by random coincidence; b) being the regional manifestations of a larger scale, northern hemispheric pattern. In this work we have opted for the second hypothesis. To verify it, we have analysed, using regime shift methods, biological and physical properties measured at different scales – from northern hemisphere to local scale (Gulf of Trieste, Mediterranean) – and have detected with different techniques the same period of shift, late 1980s, in all scales. Such synchronicity across different scales supports the idea that the local scale is linked to the hemispheric scale, and that the Mediterranean regime shift is part of a larger, northern hemisphere climate shift that happened at the end of the 1980s, which affected all European basins (explanation (b) above). We hence hypothesize that also in the other basins physical properties present a shift in this same period across different scales, from local to large, as seen in the Mediterranean Sea.

We consider also that the co-occurrence in time of regime shifts in unconnected basins provides an exceptional opportunity for assessing the relative importance of global *vs*. local drivers in shaping marine ecosystems structure. Our literature review furthermore suggests that analogous components in different basins may show similar temporal patterns. Future work should hence further address the link (if any) among systems, and among biological components of the different ecosystems, through comparative analyses, involving multiple basins and ecosystems.
